# Alcoholic–Alkali Modification of Corn Starch: A Framework for GCWS Design and Functional Tailoring

**DOI:** 10.1021/acsomega.6c03649

**Published:** 2026-08-22

**Authors:** Yung-Ho Chang, Chia-Long Lin, Xiu-Wen Xue, Jheng-Hua Lin

**Affiliations:** Department of Food and Nutrition, 63293 Providence University, Taichung 433303, Taiwan

## Abstract

Granular cold-water-soluble starch (GCWS) offers a major advancement, combining cold-water solubility with retention of native-like granule structure and paste characteristics. Among the various preparation methods, suspending starch granules in alcohol containing a trace amount of alkali—referred to as alcoholic–alkali treatment (AAT)—has been demonstrated to be particularly effective. This study systematically examined the effects of ethanol concentration (50–90%), NaOH content (1–6%), and treatment temperature (30–50 °C) on corn starch. Morphological and optical analyses revealed progressive granule shrinkage, agglomeration, fusion, and birefringence loss, with complete disruption observed in ≥3% NaOH in 50–70% ethanol. Differential scanning calorimetry showed a decline in gelatinization enthalpy under stronger alkaline conditions, culminating in the loss of ordered structures with ≥5% NaOH. Functional assessments confirmed that GCWS achieved cold-water swelling factors up to 19.4, a solubility of 12.8–29.6% at 25 °C, paste transmittance exceeding 70%, and viscosities up to 810 cP at 35 °C, outperforming untreated starch. Statistical analysis identified ethanol concentration as the primary determinant of swelling and viscosity, while NaOH content governed solubility and paste transmittance. Collectively, these findings establish optimized AAT parameters for GCWS production and provide mechanistic insights into processing–functionality relationships. The results demonstrate that precise modulation of AAT conditions enables rational design of GCWS with enhanced solubility, clarity, and viscosity, offering a robust framework for tailoring starch performance to diverse industrial applications.

## Graphical abstract

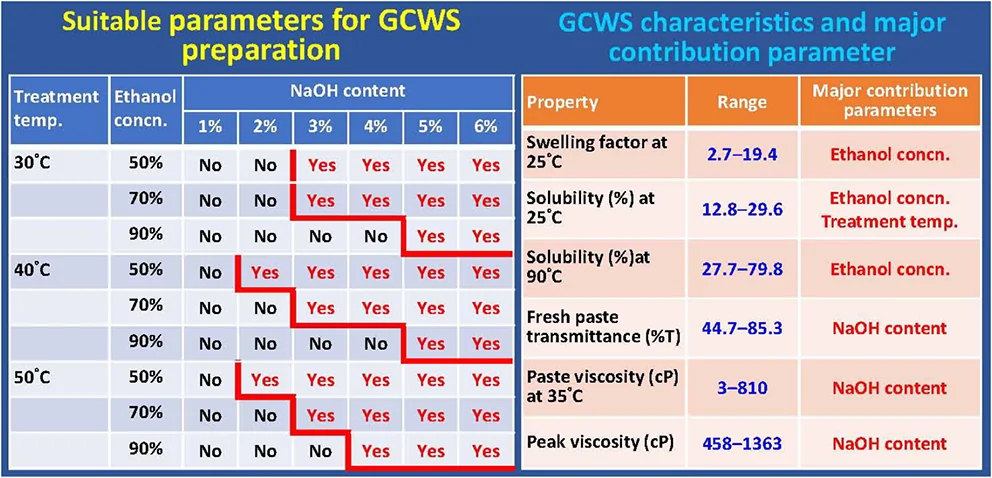

## Introduction

Corn starch, owing to its wide availability, structural versatility, and amenability to modification, has become the primary raw material in the modified starch industry. Through various modification techniques, it is able to overcome the inherent limitations of native starch, thereby achieving enhanced processing stability and functionality to support the diverse demands of the food, pharmaceutical, paper, and emerging materials industries. Starch is a semicrystalline biopolymer, is inherently insoluble in cold water, and therefore requires thermal treatment to achieve functional utility. This fundamental limitation constrains its application across diverse industries, including food, feed, and textiles. Conventional processing techniques such as drum drying, extrusion, spray drying,^1^ and ball milling^2^ have been employed to disrupt the crystalline structure of starch, yielding pregelatinized starch—commonly referred to as cold-water-soluble starch (CWS). CWS readily dissolves in cold water and has consequently broadened the industrial utility of starch.

Despite its advantages, conventional CWS suffers from particle agglomeration, reduced paste glossiness, and diminished product quality, relative to native starch formulations. These shortcomings, which primarily arise from the destruction of starch granules and the loss of native morphology during pregelatinization processes,^2,3^ limit its broader applicability and highlight the need for innovative modification strategies.

Granular cold-water-soluble starch (GCWS) represents a significant advancement in this context. GCWS is defined as starch granules that can swell in water without additional heating.^4^ GCWS dissolves in cold water while retaining a granular morphology comparable to native starch, thereby producing pastes and products with smooth texture and functional properties similar to those obtained after gelatinization. Unlike conventional CWS, which suffers from particle rupture and reduced viscosity, GCWS maintains desirable structural and sensory attributes through specialized preparation methods. Established approaches include high-temperature or high-pressure treatment in ethanol,^4,5^ polyol treatment,^6^ and alkali–alcohol treatment.^7^ Among these, the alcoholic–alkali treatment (AAT)^7^ has proven particularly effective.

AAT involves immersing starch in an ethanol solution containing alkali (typically NaOH) at room or moderately elevated temperatures, yielding GCWS with rapid hydration, high paste viscosity, and improved freeze–thaw stability. Subsequent studies have demonstrated that AAT performance is strongly influenced by ethanol and alkali concentrations as well as treatment temperature, which collectively determine the physicochemical properties of the resulting GCWS.^8,9,10^ Recent studies have demonstrated that although AAT-treated potato starch retains its granular morphology, the crystalline lattice is substantially disrupted, leading to reduced overall crystallinity and reflecting the weakening of hydrogen-bonding networks among starch chains.^11^ Mechanistically, AAT primarily targets the noncovalent interactions within starch granules: ethanol acts as a dehydrating medium that destabilizes interchain hydrogen bonds, while alkali facilitates the loosening of double-helical structures by increasing electrostatic repulsion. This cooperative action results in the collapse of ordered crystalline domains without cleavage of covalent glycosidic linkages or formation of new chemical bonds.

Meanwhile, novel physical and enzyme-assisted techniques—such as pulsed electric field treatment combined with enzymatic and alcoholic–alkaline processing^12^ and dual modification using cold plasma and ozonation^13^—have demonstrated potential for enhancing CWS or GCWS functionality. However, these approaches involve complex procedures and reactions, posing challenges in process control and cost management. By contrast, the strategic refinement and precise modulation of established processes such as AAT constitute an equally critical pathway for advancing GCWS technology to meet industrial demands.

Collectively, these findings establish AAT as a physical modification technique that selectively disrupts hydrogen-bonding interactions, thereby providing a versatile and effective pathway for the production of GCWS. Although prior studies have examined the effects of individual parameters in alcoholic–alkali treatment (AAT), comprehensive evaluation of their combined influence remains scarce. Native starches inherently exhibit poor cold-water solubility and limited functional versatility, which restricts their direct application in food and industrial systems. Conventional modification approaches often rely on high-temperature processing or chemical derivatization, which may compromise granular integrity or increase production costs. In this context, AAT represents a distinctive and relatively mild strategy that enables the preparation of granular cold-water-soluble (GCWS) starch while preserving granule morphology. The present study systematically investigates three pivotal parameters—ethanol concentration (50–90%), NaOH content (1–6%), and treatment temperature (30–50 °C)—to address this research gap. By integrating mechanistic characterization of swelling factor, cold-water solubility, paste transmittance, and viscosity, this work establishes a predictive framework that clarifies how each parameter contributes to GCWS functionality. The findings not only demonstrate why AAT is required but also provide a versatile foundation for rationally designing GCWS properties to meet diverse industrial application needs.

## Materials and Methods

### Materials

Corn starch used in this study was obtained from Liou He Industrial Co., Ltd. (New Taipei City, Taiwan) and was sourced from Amyral (South Africa). All chemical reagents employed were of analytical grade.

### Preparation of AAT Starch

AAT-treated starch was prepared following the method described by Chen and Jane (1994),^8^ with modifications. Starch (150 g, db) was weighed and suspended in 600 mL of ethanol solution (50%, 70%, or 90%, v/v) in a 1 L three-necked flask. The suspension was thoroughly stirred to ensure complete dispersion of starch granules, after which 1–6 mL of concentrated (50%, w/w) NaOH solution per 100 mL of ethanol solution was added. In this study, the unit (%) for NaOH content denotes the volume (mL) of concentrated NaOH solution per 100 mL of ethanol solution. The mixture was reacted in a water bath at 30, 40, or 50 °C with continuous stirring at 50 rpm for 1 h. After treatment, the starch suspension was filtered through a Whatman No. 4 filter paper and washed with 300 mL of 90% ethanol solution. The starch was then resuspended in 300 mL of 90% ethanol and neutralized with a 3 M HCl solution. Following neutralization, the starch was washed three times with 300 mL of 90% ethanol and once with 300 mL of 95% ethanol and then filtered. The resulting starch was oven-dried at 80 °C for 3 h, pulverized, passed through an 80-mesh screen (aperture diameter 180 μm), and sealed in a container for storage at room temperature until use. The recovery of AAT-treated corn starches was consistently above 95%, with the minor loss mainly arising from the removal of alkali and repeated washing during the recovery process.

### Morphology of Starch Granules

The morphology of starch granules was observed by using a scanning electron microscope (SEM, JSM-7800F, JEOL, Akishima, Tokyo, Japan). The starch was affixed to the observation stub with double-sided adhesive tape. After drying and gold coating, the starch was observed at an accelerating voltage of 3.0 kV.

### Light and Polar-Light Microscopy of Starch Granules

The swelling behavior and birefringence of starch granules at room temperature were examined by using light and polarizing microscopy (BX41, Olympus, Tokyo, Japan), respectively. Starch granules were suspended in deionized water at room temperature and mounted on glass slides for observation. Birefringence was further evaluated with the same microscope equipped with a polarizing filter.

### Gelatinization Enthalpy Change

Starch samples (150 mg, db) were accurately weighed into sample pans, to which deionized water was subsequently added at a water-to-starch ratio of 3:1 (w/w). The pans were sealed and allowed to stand at room temperature overnight to ensure complete hydration. After equilibration, the samples were analyzed using a differential scanning calorimeter (Micro DSC VII, Setaram, France). Each pan was held at 25 °C for 3 min and then heated to 115 °C at a rate of 1.2 °C/min. The resulting endothermic curves were used to determine the gelatinization enthalpy change (*ΔH*).

### X-ray Diffraction

Wide-angle X-ray diffraction (XRD) analysis of starch was conducted following the procedure described by Lin et al. (2022).^14^ Prior to measurement, the starch samples were equilibrated under saturated relative humidity. Diffraction patterns were recorded over a Bragg angle (2θ) range of 5°–40°. The diffractometer (Shimadzu XRD-6000, Kratos Analytical Inc., Kyoto, Japan) was operated at 40 kV and 30 mA, with a scanning rate of 1.2°/min.

### Swelling Factor

The method described by Li et al. (2015)^15^ was used to determine the swelling factor of the samples in this study. 100 mg (dry weight) of samples was weighed into a test tube, and 5 mL of deionized water was added. After shaking in a water bath at 25 °C for 30 min, 0.5 mL of blue dextrin solution (5 mg/mL) was added and mixed thoroughly. The tube was then centrifuged at 1,500 × *g* for 5 min. The absorbance of the supernatant from the sample tubes (AS) and reference tube (AR, contained no sample) at 620 nm was measured by a spectrophotometer. The swelling factor of the samples was calculated as follows:

$$\mathrm{Swelling factor}\left(v/v\right)=1+\left(\frac{7700}{W}\times \frac{A_{S}-A_{R}}{A_{S}}\right)$$

where W is the dry weight of the sample in mg.

### Solubility

The solubility of the samples was determined based on the method of Chang et al. (2010)^16^ with slight modifications. Starch samples (1 g, dry basis) were dispersed in 100 mL of deionized water and shaken in a water bath at 25 or 90 °C for 30 min. The resulting suspension was centrifuged at 1,200 × *g* for 15 min. The supernatant was carefully decanted, transferred to a preweighed dish, and dried in an oven at 130 °C to constant weight. Solubility (%) was calculated using the following equation:

$$\mathrm{Solubility}(\%)=\frac{\mathrm{grams of solids in supernatant}}{\mathrm{grams of sample}}\times 100$$

### Paste Transmittance

The paste transmittance of the samples was assessed following the method of Bello-Pérez et al. (1999).^17^ A 1% (w/w) starch suspension (5 mL) was transferred into spiral tubes and heated in a boiling water bath for 30 min, with shaking every 5 min to ensure uniform gelatinization. After heating, the tubes were cooled to room temperature for 10 min and subsequently stored at 4 °C for 7 days. Paste transmittance was expressed as transmittance (%T), measured at 650 cm^–1^ using a spectrophotometer (Genesys 20, Thermo Scientific, USA) for both freshly prepared and 7-day-stored starch pastes.

### Paste Viscosity

The paste viscosity of the samples was analyzed using a Rapid Visco Analyzer (RVA TecMaster; Newport Scientific, Warriewood, Australia). A 7% (w/w) starch suspension with a total weight of 28 g was prepared by accurately weighing the required amount of starch and slowly dispersing it into the water in the aluminum test canister under continuous stirring to ensure complete hydration without lump formation. The measurement program was set as follows: the sample was first equilibrated at 35 °C for 1 min, then heated to 95 °C at a rate of 6 °C/min, and subsequently held at 95 °C for 5 min. Throughout the heating cycle, viscosity was recorded at a stirring speed of 160 rpm. The viscosity at 35 °C during the initial holding period and the peak viscosity (PV) observed during heating were documented.

### Statistical Analysis

This study employed a completely randomized design, with all samples analyzed in triplicate. Differences among the obtained data were evaluated using Duncan’s multiple range test provided by Statistical Analysis System (SAS Institute, USA). The general linear model (GLM) procedure was applied to assess the contributions of alcoholic–alkali treatment conditions to the swelling properties, paste transmittance, and viscosity of GCWS. The contribution degree (%) of each variable to the overall variability was calculated based on the sum of squares derived from the analysis of variance.

## Results and Discussion

### Granule Morphology

Table 1 presents the SEM observations of corn starch granules subjected to the AAT under varying treatment conditions. Untreated corn starch granules exhibited a polygonal morphology, were well-dispersed, and predominantly possessed smooth surfaces, although some granules displayed surface pores. Following AAT, the starch granules underwent distinct morphological alterations, including shrinkage, agglomeration, and fusion, depending on the treatment condition (Supporting Information Figures 1–3). As revealed through SEM examination, the granular morphology of starch was observed to be classified into four categories: unchanged, granule shrinkage (S), granule shrinkage accompanied by agglomeration (S+A), and granule shrinkage accompanied by fusion (S+F).

**Table 1:**
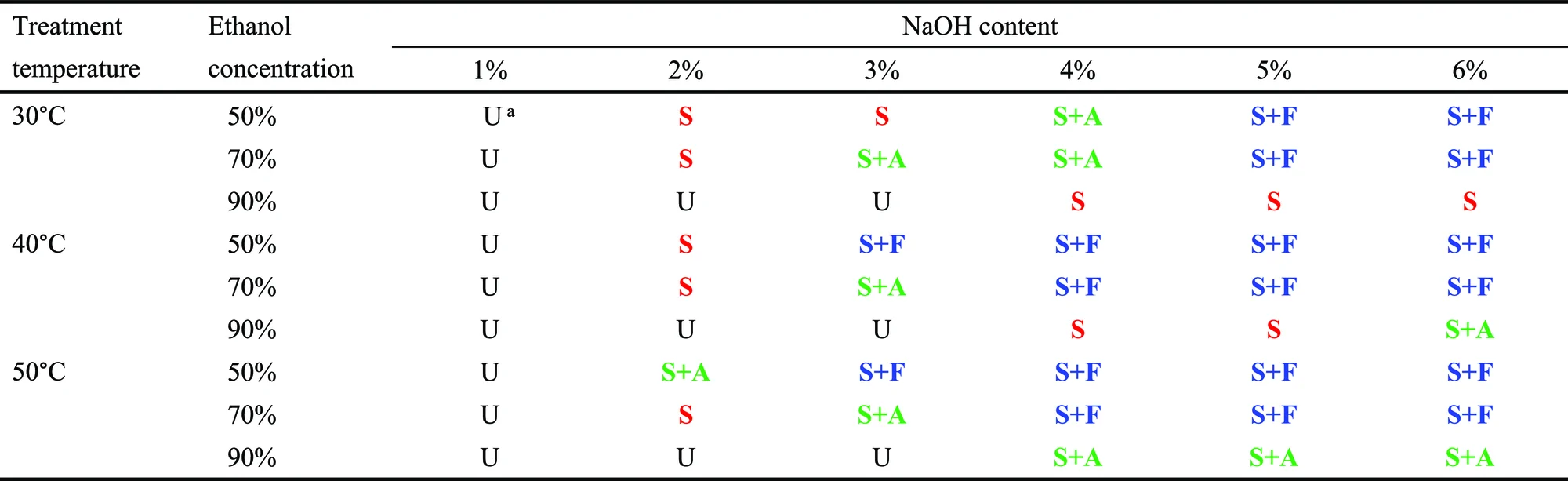
Granule Morphology Observed by Scanning Electron Micrography of Alcoholic–Alkaline-Treated Starches^a^

a Typical morphology of starch granules together with their abbreviations, bar = 10 μm.
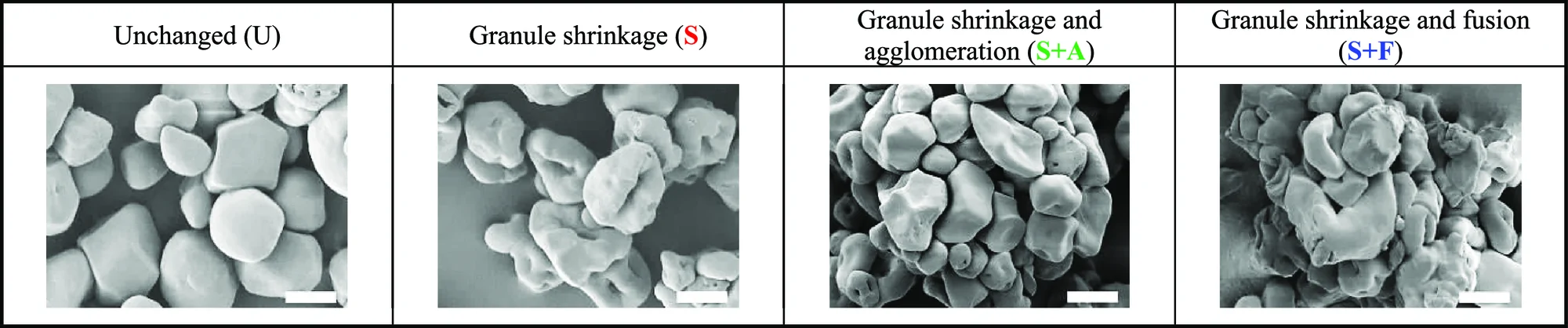

These morphological transitions are indicative of progressive structural modifications occurring under applied treatment conditions. The unchanged granules suggest that certain starch populations retained their native resistance to processing stress, whereas shrinkage alone (S) reflects the partial dehydration and collapse of the granule surface. The occurrence of agglomeration (S+A) implies that weakened granules tend to adhere to one another, possibly as a result of increased molecular mobility or partial gelatinization during AAT. Fusion (S+F), by contrast, denotes a more advanced stage of molecular moving among starch molecules from adjacent granules, wherein the granules progressively lose their discrete boundaries and coalesce into continuous structures. Taken together, these observations highlight the dynamic nature of starch granule responses to external stimuli and suggest that the degrees of shrinkage, agglomeration, and fusion may serve as morphological indicators of processing intensity and structural integrity. Such insights are valuable for understanding starch functionality in food systems and for optimizing processing conditions to achieve the desired textural and physicochemical properties.

Ethanol solutions containing 1% NaOH did not induce noticeable morphological alterations relative to untreated starch granules. In contrast, exposure to ethanol solutions containing 2% and 3% NaOH resulted in a morphology that was markedly dependent on ethanol concentration. Treatments with 50% and 70% ethanol produced pronounced granule shrinkage, shrinkage accompanied by agglomeration, and, in certain instances, shrinkage with fusion. However, when the ethanol concentration was elevated to 90%, granules treated with 2% and 3% NaOH exhibited only negligible morphological modifications. Furthermore, starch granules treated with ethanol solutions containing 3–6% NaOH consistently demonstrated substantial shrinkage, often accompanied by agglomeration or fusion, regardless of ethanol concentration. This indicates that granule deformation is exacerbated under conditions of elevated NaOH content, reduced ethanol concentration, or increased treatment temperature.

In summary, the extent of morphological disruption in AAT-treated corn starch granules is governed by a synergistic interplay among the NaOH content, ethanol concentration, and thermal conditions. Higher NaOH levels, lower ethanol concentrations, and elevated temperatures collectively contribute to more pronounced granule shrinkage, coagulation, and fusion.

### Granule Swelling and Birefringence

In this study, the swelling behavior of starch granules suspended in water at room temperature was observed by using a light microscope. In addition, their birefringence was analyzed with a polarizing attachment, and the corresponding results are summarized in Table 2. Untreated corn starch granules maintained intact polygonal shapes without noticeable swelling and exhibited distinct birefringence. Following AAT, the granules displayed diverse morphological features under microscopic observation, strongly influenced by the treatment conditions (Supporting Information Figures 4–6). On the basis of the extent of swelling and the changes in birefringence, the granules were classified into three categories: unchanged (U), retaining their original morphology with no obvious swelling and preserved birefringence; partially swollen with partial loss of birefringence (P), showing localized swelling accompanied by a reduction in birefringence; and completely swollen with complete loss of birefringence (C), in which all granules were markedly swollen and birefringence was entirely absent.

**Table 2:**
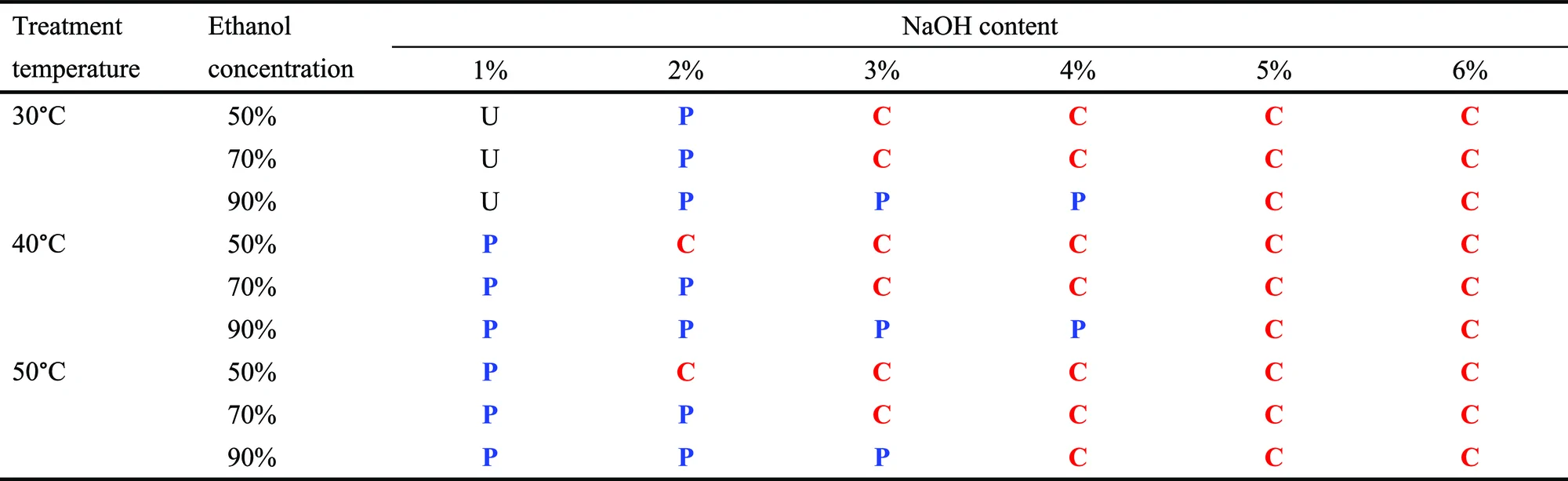
Granule Morphology and Birefringence of Alcoholic–Alkaline-Treated Starches Observed by Light (LM) and Polarized-Light (PLM) Micrography^a^

a Typical morphology and birefringence of starch granules together with their abbreviations, bar = 20 μm.
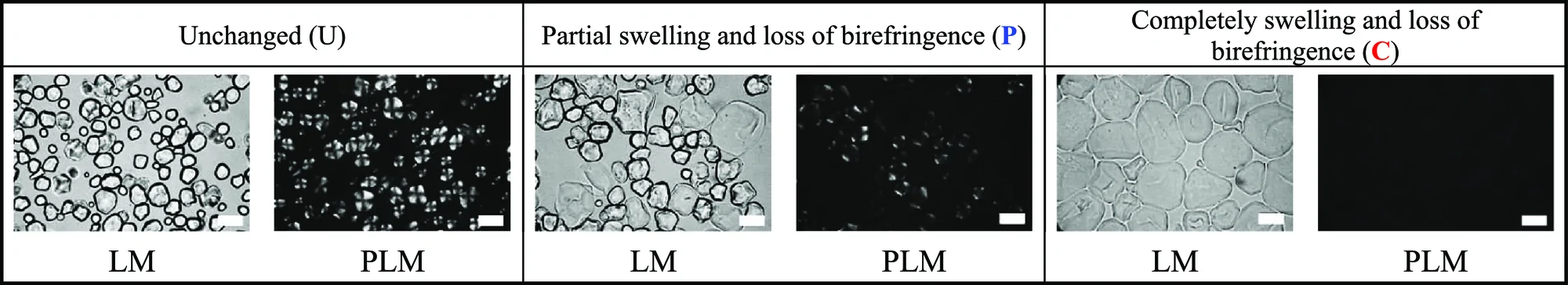

When starch was treated with 1% NaOH in ethanol solution at 30 °C, the granules exhibited no significant changes in swelling behavior or birefringence in water, remaining consistent with those observed in untreated starch. However, when the treatment temperature was increased to 40 and 50 °C, some AAT starch granules showed partial swelling accompanied by loss of birefringence. Increasing the NaOH content to 2% resulted in AAT starch that, when suspended in water, displayed either partial swelling with loss of birefringence or complete swelling with disappearance of birefringence. Under conditions of a 50% ethanol solution at 40 or 50 °C, all starch granules in water were swollen and lost birefringence. When the NaOH content was raised to 3% and 4%, starch granules obtained under all conditions, except those that were treated in a 90% ethanol solution, were completely swollen and lost birefringence in water. Upon further increasing the NaOH content to 5% and 6%, the resulting AAT starch granules, regardless of ethanol concentration or treatment temperature, were uniformly swollen and exhibited complete loss of birefringence.

Results from Tables 1 and 2 indicate that corn starch subjected to AAT undergoes morphological alterations accompanied by partial or complete swelling in water at ambient temperature. The swollen granules concurrently exhibit a loss of birefringence. These phenomena are strongly dependent on the specific AAT conditions—including ethanol concentration, NaOH content, and treatment temperature—with NaOH content exerting the most pronounced influence. Furthermore, elevated treatment temperatures and reduced ethanol concentrations markedly intensify the structural modifications of the starch granules.

### Gelatinization Enthalpy Change

The gelatinization enthalpy change of untreated corn starch was 12.8 J/g (Table 3). When starch was treated with ethanol containing 1% NaOH, the gelatinization enthalpy change of the resulting AAT starch ranged between 12.4 and 13.0 J/g, showing no significant difference from untreated starch and indicating that the content of ordered structures was not markedly altered.^18^ In contrast, treatment with ethanol containing 2% NaOH produced AAT starch with gelatinization enthalpy change values of 10.7–12.2 J/g when obtained from 70% and 90% ethanol solutions, which were slightly lower than that of untreated starch. Notably, AAT starch treated in a 50% ethanol solution exhibited no detectable gelatinization enthalpy change, suggesting that the ordered structures had been disrupted by the AAT.

**Table 3:** Gelatinization Enthalpy Change (J/g) of Alcoholic–Alkaline-Treated Starches^a^

		NaOH content					
Treatment temperature	Ethanol concentration	1%	2%	3%	4%	5%	6%
30 °C	50%	13.0 ± 0.3	**ND** ^b^	**ND**	**ND**	**ND**	**ND**
	70%	12.7 ± 0.1	11.8 ± 0.2	**ND**	**ND**	**ND**	**ND**
	90%	12.9 ± 0.2	11.1 ± 0.1	9.7 ± 0.1	6.1 ± 0.1	**ND**	**ND**
40 °C	50%	12.4 ± 0.1	**ND**	**ND**	**ND**	**ND**	**ND**
	70%	13.0 ± 0.3	10.9 ± 0.1	**ND**	**ND**	**ND**	**ND**
	90%	12.8 ± 0.1	11.9 ± 0.2	9.0 ± 0.1	3.5 ± 0.1	**ND**	**ND**
50 °C	50%	12.6 ± 0.3	**ND**	**ND**	**ND**	**ND**	**ND**
	70%	12.9 ± 0.2	12.2 ± 0.1	**ND**	**ND**	**ND**	**ND**
	90%	13.0 ± 0.1	10.7 ± 0.2	2.0 ± 0.1	**ND**	**ND**	**ND**

a The gelatinization enthalpy change of untreated corn starch is 12.8 ± 0.8 J/g.

b Not detected.

Further treatment with 3% and 4% NaOH in 50% and 70% ethanol solutions also resulted in AAT starch with no gelatinization enthalpy change. Only when treated in a 90% ethanol solution did the gelatinization enthalpy change range between 2.0 and 9.7 J/g, which was significantly lower than that of untreated starch. Moreover, at the same NaOH content, the gelatinization enthalpy change decreased progressively with increasing treatment temperature. When the NaOH content was increased to 5% and 6%, regardless of ethanol concentration or treatment temperature, the resulting AAT starch exhibited no gelatinization enthalpy change, indicating that the ordered structures had been destroyed.

Figure 1 presents the X-ray diffraction (XRD) patterns of corn starch subjected to alkali–alcohol treatment in 50% ethanol containing 1%, 3%, or 5% NaOH at 30 °C. Untreated corn starch exhibited a typical A-type diffraction pattern, and the starch treated with 1% NaOH in ethanol solution showed no appreciable difference from the untreated sample, indicating that its crystalline structure was largely preserved. In contrast, starches treated with 3% and 5% NaOH in ethanol solutions displayed only weak diffraction peaks, or even an absence of discernible crystalline peaks, suggesting that most of the crystalline structure was disrupted or completely lost. These findings are consistent with the results for gelatinization enthalpy values of starch granules shown in Table 3, collectively demonstrating that alkali–alcohol treatment induces varying degrees of disruption to the ordered or crystalline structure of starch depending on treatment conditions.

**Figure 1:**
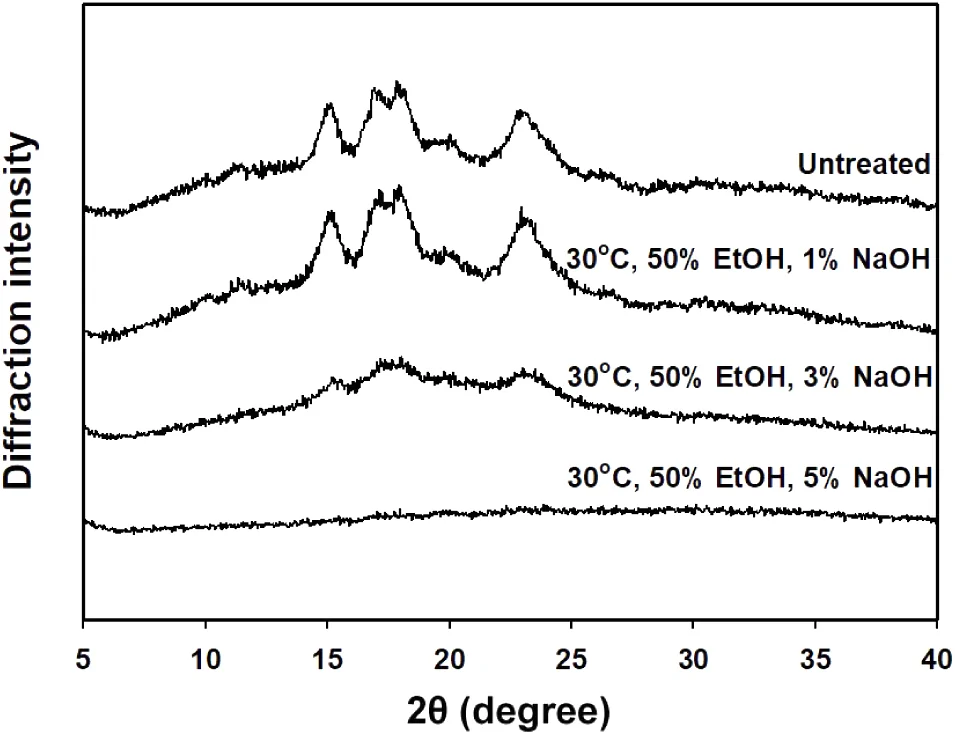
X-ray diffraction patterns of untreated and alcoholic–alkali-treated corn starches.

In summary, the gelatinization enthalpy change of AAT starch is highly dependent on treatment conditions. Factors such as ethanol concentration, NaOH content, and treatment temperature play critical roles in determining the extent of ordered structures within the starch. At a low NaOH content of 1%, the ordered structures remained largely intact. In contrast, when the NaOH content reached 5% or higher, these structures were completely disrupted, resulting in the absence of detectable gelatinization enthalpy change. For AAT starch treated with intermediate NaOH contents (3–4%), the gelatinization enthalpy change was markedly influenced by ethanol content and treatment temperature, exhibiting a clear trend in which lower ethanol concentrations combined with higher treatment temperatures led to greater disruption of the ordered structures.

### Suitable AAT Parameters for GCWS Production

Zhao et al. (2023)^19^ reported that in an alcoholic–alkali system, strong alkali acts on the hydroxyl groups of starch molecules, imparting negative charges that disrupt intermolecular hydrogen bonds. Consequently, the double-helical structures are converted into single helices, thereby facilitating the expansion of starch granules. Conversely, the presence of ethanol suppresses starch swelling, which helps preserve the integrity of the granular structure. In this study, ethanol concentration, NaOH content, and treatment temperature potentially applicable to alcoholic–alkali treatment (AAT) were selected for starch modification. The suitable conditions for preparing GCWS were screened based on granule morphology, granular deformation and birefringence, and enthalpy change during gelatinization. The resulting starch granules were in a semistable state and exhibited cold-water solubility and thus defined as GCWS. The results presented in Table 1 (granule morphology), Table 2 (granular deformation and birefringence in water at room temperature), and Table 3 (gelatinization enthalpy change) consistently demonstrate granule deformation, swelling in cold water, disappearance of birefringence, and loss of ordered structures under varying treatment conditions. These findings are consistent with the aforementioned characteristics of GCWS.

Based on these findings, the suitable parameters for preparing GCWS by AAT are summarized in Table 4. Ethanol solutions containing only 1% NaOH are ineffective for GCWS production. With 2% NaOH, GCWS can be obtained only in 50% ethanol at treatment temperatures of 40 and 50 °C; treatment at 30 °C produces incomplete swelling and birefringence loss and is therefore not considered effective. At NaOH contents of 3–4%, GCWS can be prepared in both 50% and 70% ethanol solutions. In contrast, 90% ethanol restricts granule swelling and molecular structural changes, allowing GCWS formation only with 4% NaOH at 50 °C. When NaOH content reaches 5% or higher, the removal of ordered structures is facilitated, enabling granules to swell in cold water and thereby form GCWS.

**Table 4:**
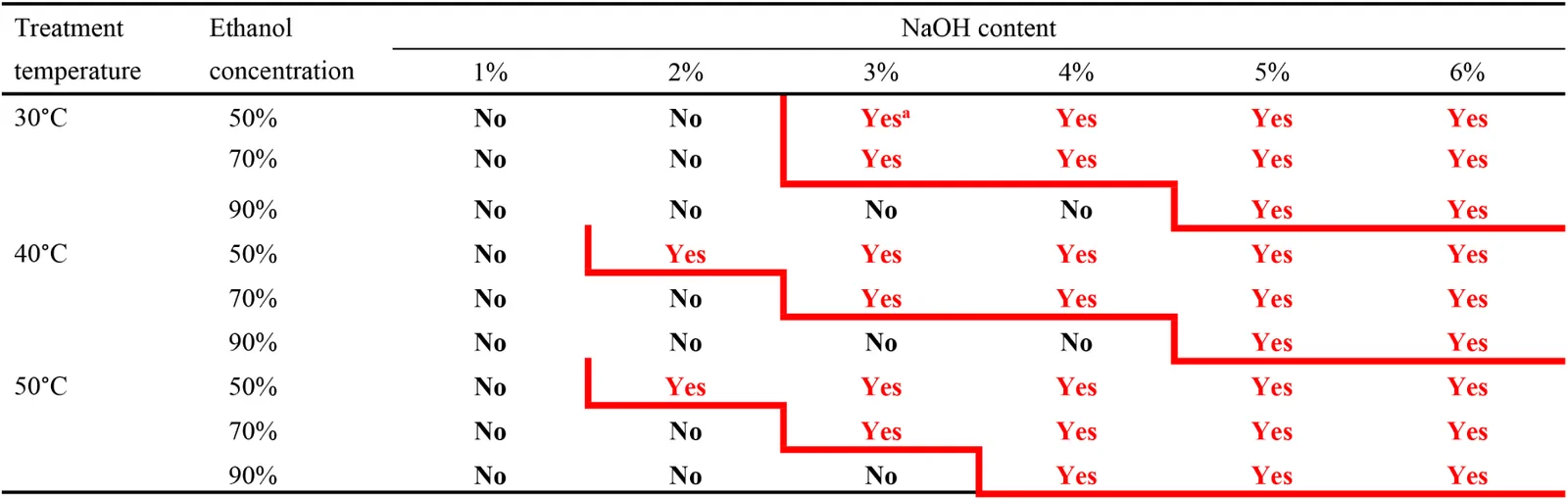
Suitable AAT Parameters for Granular Cold-Water-Soluble Starches (GCWS) Production

a Suitable conditions for GCWS production refer to the fact that the treated starch has no gelatinization thermal peak, and all particles can swell in cold water and lose birefringence.

To further clarify the influence of treatment conditions on GCWS properties, swelling factor, solubility, paste clarity, and paste viscosity were compared across the samples, and statistical analyses were conducted to evaluate the relative contributions of the treatment parameters to the GCWS characteristics.

### Swelling Factor of GCWS

Figure 2A illustrates the swelling factor of GCWS prepared under different AAT conditions. The swelling factor reflects the extent to which starch granules expand in volume during water absorption. Untreated corn starch shows a swelling factor of only 1.5 at 25 °C due to the absence of gelatinization; however, at 90 °C, gelatinization occurs and the granules absorb large amounts of water, increasing the swelling factor to 17.3.

**Figure 2:**
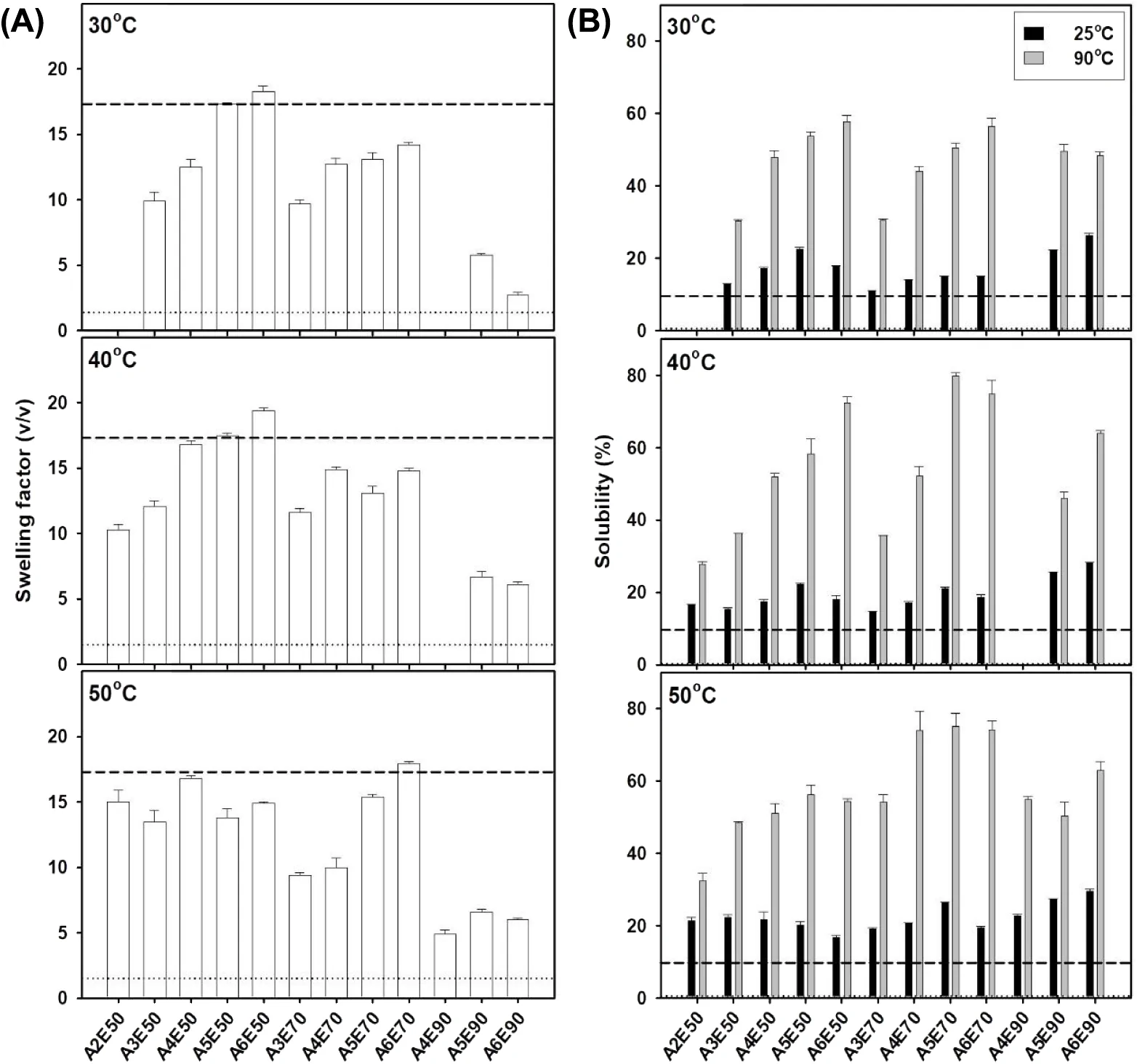
Swelling factor at 25 °C (A) and solubility at 25 and 90 °C (B) of granular cold-water-soluble starches (GCWS). In the sample names, “A” denotes NaOH content and “E” denotes ethanol concentration, while the numbers following A and E indicate the respective concentrations. The dotted (···) and short-dash (− – −) lines represent the swelling factor (A) and solubility (B) of untreated starch at 25 and 90 °C, respectively.

GCWS samples prepared via AAT exhibit swelling factors at 25 °C ranging from 2.7 to 19.4, confirming their cold-water swelling capability. The swelling properties, however, vary considerably with treatment conditions. For GCWS prepared at 50 °C in 50% ethanol, the swelling factor shows no clear relationship with NaOH content. In contrast, GCWS produced in 50% and 70% ethanol demonstrates a pronounced increase in swelling factor with rising NaOH content. GCWS prepared in 90% ethanol consistently shows much lower swelling factors compared with those produced in 50% and 70% ethanol. Moreover, no systematic trend is observed across different treatment temperatures, suggesting that temperature exerts a less consistent influence on swelling behavior.

### Solubility of GCWS

Starch solubility reflects the proportion of amylose released during gelatinization or starch fragments generated from granule disintegration that dissolve in the aqueous phase. In this study, untreated corn starch exhibited extremely limited solubility in cold water, with only 0.1% at 25 °C; upon heating to 90 °C, solubility increased to 9.8%. As a restricted-swelling starch, corn starch shows lower amylose leaching and granule disintegration compared with high-swelling starches (e.g., potato starch), and its solubility remains below 10% even under high-temperature conditions.

By contrast, GCWS produced via AAT displayed markedly higher solubility, ranging from 12.8 to 29.6% at 25 °C (Figure 2B), confirming its cold-water solubility. For GCWS prepared in 50% and 70% ethanol solutions, solubility increased with NaOH content, though the rate plateaued or slightly declined at 5–6% NaOH. GCWS prepared in 90% ethanol showed only a slight continuous increase in solubility with NaOH content.

At 90 °C, GCWS solubility further increased to 27.7–79.8%, reflecting enhanced amylose leaching and granule disintegration. Similar to cold-water conditions, solubility in 50% and 70% ethanol rose with NaOH content but diminished at 6% NaOH, while GCWS prepared in 90% ethanol exhibited no clear trend.

### Paste Transmittance of GCWS

Figure 3A illustrates the changes in paste transmittance of GCWS samples. The 1% paste of untreated corn starch exhibited an initial transmittance (T%) of only 31.0%, which decreased further to 18.6% after 7 days of storage, reflecting the typical retrogradation and loss of optical clarity observed in low-transparency starches. In contrast, GCWS prepared under various AAT conditions showed markedly higher initial transmittance values ranging from 44.7% to 85.3%, indicating that AAT effectively enhanced the optical transparency of corn starch. After storage at 4 °C for 7 days, GCWS transmittance remained within 25.3–84.7%, consistently higher than that of untreated starch. Moreover, GCWS samples with initially high transmittance (>70%) exhibited relatively minor changes after storage, whereas those with lower initial values (<70%) showed more pronounced decreases.

**Figure 3:**
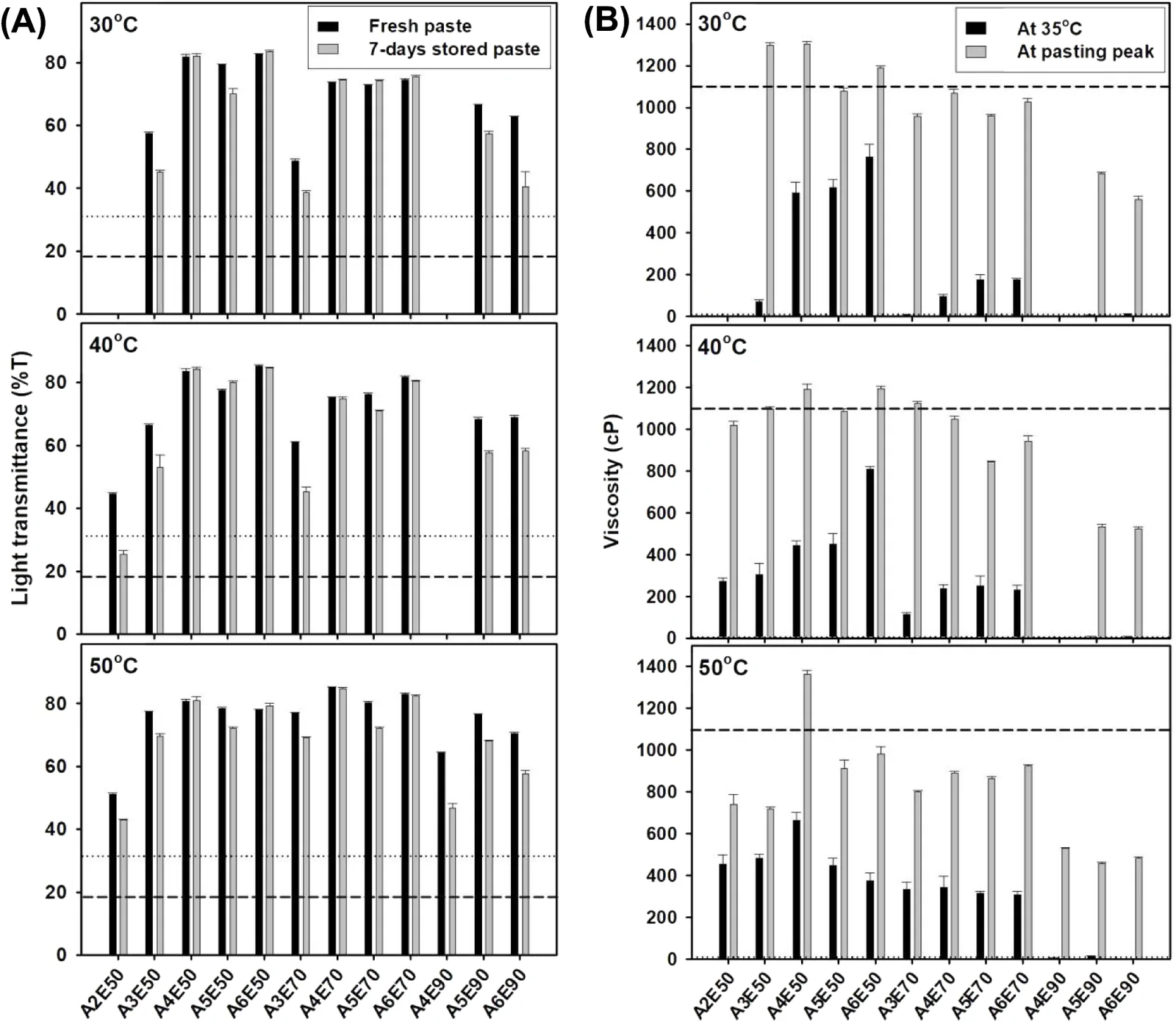
Light transmittance (A) and viscosity (B) of pastes prepared from granular cold-water-soluble starches (GCWS). In the sample names, “A” denotes NaOH content and “E” denotes ethanol concentration, while the numbers following A and E indicate the respective concentrations. The dotted (···) and short-dash (− – −) lines are the paste transmittance of fresh and 7-day-stored pastes of untreated starch (A), and paste viscosity at 35 °C and peak viscosity of untreated starch (B), respectively.

Among GCWS samples prepared under different ethanol concentrations, those produced in 50% and 70% ethanol systems exhibited increasing transmittance with rising NaOH content when NaOH was below 4%. Once NaOH content reached 4–6%, the differences in transmittance became minimal, with all values exceeding 70%. This suggests that starch molecules had undergone sufficient swelling and that intermolecular entanglements were largely removed, resulting in optical properties no longer strongly affected by NaOH content. In contrast, GCWS prepared in 90% ethanol generally exhibited lower transmittance, likely due to restricted swelling and limited molecular expansion under high ethanol concentrations, which reduced optical homogeneity.

### Paste Viscosity of GCWS

Untreated corn starch exhibited almost no measurable viscosity at 35 °C in the Rapid Visco Analyzer (RVA), as the granules had not yet gelatinized. In contrast, GCWS samples displayed substantial viscosity at 35 °C, ranging from 3 to 810 cP (Figure 3B), clearly demonstrating their cold-water solubility.

For GCWS prepared in 50% and 70% ethanol at treatment temperatures of 30 and 40 °C, viscosity at 35 °C increased with rising NaOH content, reaching approximately 810 cP. When the treatment temperature was raised to 50 °C, viscosity differences among GCWS samples narrowed to 307–665 cP. GCWS prepared in 50% ethanol exhibited a slightly higher viscosity than those prepared in 70% ethanol, although no consistent trend was observed with respect to NaOH content. In contrast, GCWS prepared in 90% ethanol showed extremely low viscosities at 35 °C (3–13 cP), regardless of treatment temperature.

The PV of untreated corn starch was 1103 cP, whereas GCWS samples’ ranged from 458 to 1363 cP. GCWS prepared in 50% and 70% ethanol at 30 and 40 °C exhibited relatively high PV values (843–1303 cP), again without a clear trend across NaOH content. The highest PV (1363 cP) was observed in GCWS prepared with 4% NaOH in 50% ethanol. GCWS prepared at 50 °C showed lower PV values (717–980 cP) than those prepared at lower temperatures, and GCWS produced in 90% ethanol consistently exhibited lower PV than samples prepared in 50% and 70% ethanol.

In total, GCWS exhibited substantial viscosity at 35 °C, with the highest value reaching 73% of the PV of untreated starch. The PV of GCWS corresponded to 42–124% of that of untreated starch, indicating that GCWS retained the inherent pasting ability of native starch granules. These results demonstrate that adjusting AAT processing conditions effectively modulates pasting viscosity, thereby enhancing the functional flexibility of the material.

### Contribution of AAT Parameters to GCWS Characteristics

The contribution analysis consolidates and quantifies the effects of ethanol concentration, NaOH content, and treatment temperature on GCWS properties, enhancing coherence and interpretability. It demonstrates how these parameters regulate starch granule swelling, molecular mobility, and structural disruption, thereby shaping GCWS functionality and providing a scientific basis for designing processing conditions and reinforcing the application value of AAT in starch modification. Results of the statistical analysis (Table 5) demonstrate that the AAT parameters—including ethanol concentration, NaOH content, and treatment temperature—exerted significant effects on the swelling factor, solubility, paste transmittance, and paste viscosity of GCWS. However, the magnitude of influence varied across different response variables. The swelling factor of GCWS was predominantly governed by ethanol concentration, which contributed 68.9% of the total effect. Given that ethanol concentration is closely associated with the restriction of granule swelling and molecular mobility or rearrangement during AAT, this finding suggests that enhanced molecular mobility or rearrangement induced by AAT facilitates the swelling of GCWS granules.

**Table 5:** Contribution Degree (%) of Ethanol Concentration (E), NaOH Content (A), and Treatment Temperature (T) to Swelling Factor, Solubility, and Paste Light Transmittance of GCWS

Properties	E	A	T
Swelling factor	**69.8**	18.5	1.3
Solubility			
25 °C	**20.6**	11.8	**20.4**
90 °C	10.9	**54.1**	13.7
Light transmittance			
Fresh paste	16.3	**50.8**	11.2
7-day stored paste	28.8	**52.7**	5.6
Paste viscosity			
At 35 °C	**70.6**	11.2	3.6
At pasting peak	**57.1**	6.4	8.7

With respect to solubility, the contributions of processing parameters to GCWS solubility at 25 °C (cold-water solubility) were relatively balanced, with ethanol concentration (20.6%) and treatment temperature (20.4%) exerting slightly greater influence than NaOH content (11.8%). In contrast, GCWS solubility at 90 °C was primarily determined by NaOH content, which accounted for 54.1% of the contribution. These results highlight the distinct effects of AAT preparation conditions on GCWS solubility at 25 and 90 °C. At 25 °C, GCWS granules absorbed water, swelled, and released partial molecular components; however, swelling under cold-water conditions rarely led to excessive expansion or granule disintegration. Consequently, differences in cold-water solubility among samples were relatively limited (12.2–29.6%), indicating minimal influence of preparation conditions. By contrast, at 90 °C, GCWS granules underwent pronounced swelling, resulting in excessive expansion or disintegration and thereby producing a substantial increase in solubility. The differences among samples were considerably greater (27.7–79.8%), underscoring the stronger impact of preparation parameters under hot-water conditions. Compared with untreated starch, GCWS exhibited higher solubility in both cold and hot water (Figure 2B), confirming that AAT is more effective than simple heating in disrupting ordered structures and intermolecular entanglements within starch granules. This effect is primarily attributable to NaOH content.

The paste transmittance of GCWS, both freshly prepared and after 7 days of storage, was likewise mainly influenced by alkali content, with contribution rates of 50.8% and 52.7%, respectively. Paste transmittance is strongly dependent on the degree of ordered structure and molecular entanglement within starch. Consistent with the solubility results at 90 °C, higher alkali content promoted more extensive disruption of ordered structures, thereby enhancing paste clarity.

For paste viscosity, both the viscosity at 35 °C and the PV of 7% GCWS suspensions were primarily affected by ethanol concentration, with contribution rates of 70.6% and 57.1%, respectively. The GCWS samples analyzed in this study were prepared under AAT conditions selected based on granule swelling, loss of birefringence, and thermal properties (Table 4), yielding materials with extensively disrupted ordered structures and cold-water solubility. Since ethanol concentration influences granule swelling and molecular mobility during AAT, its high contribution to paste viscosity indicates that GCWS viscosity is closely linked to the extent of granule swelling and molecular mobility occurring during processing.

### Effects of AAT Parameters on GCWS Properties

Jane et al. (1986)^4^ defined GCWS as starch granules that disrupt the native double-helix crystalline packing and convert it into single helices. This transformation yields granules that remain intact but become metastable and soluble in cold water without conventional gelatinization. GCWS can undergo gelatinization without heating, simply by the addition of sufficient water while retaining pasting properties similar to those of native starch. This unique feature, which is not achievable with conventional pregelatinized starch, is of considerable importance in enhancing both the convenience and the sensory quality of instant foods.

Based on the overall results of GCWS characteristics in this study, it was found that GCWS exhibited remarkable swelling capacity at room temperature, with swelling factors comparable to or even exceeding those of native corn starch gelatinized at 90 °C (Figure 2A). The swelling factor of GCWS was primarily governed by ethanol concentration, with a contribution rate of 68.9%, indicating that ethanol concentration plays a critical role in restricting granule swelling and molecular mobility. In 90% ethanol, swelling was strongly suppressed, whereas in 50% and 70% ethanol systems, NaOH content became a secondary factor, with increasing concentrations markedly enhancing granule swelling.

GCWS demonstrated significantly higher solubility at 25 °C than native starch (Figure 2B), confirming that alcoholic–alkali treatment disrupts ordered structures more effectively than heating alone. Cold-water solubility was influenced slightly more by ethanol concentration (20.6%) and treatment temperature (20.4%) than by NaOH content (11.8%), reflecting the limited differences under partial swelling conditions. Conversely, at 90 °C, NaOH content was the dominant factor, with a contribution rate of 54.1%. Excessive swelling and granule disintegration led to a substantial increase in solubility, with values ranging from 27.7% to 79.8%.

According to Achille et al. (2007),^20^ corn starch is classified as a low-clarity starch (11–15%), whereas tapioca and potato starches exhibit much higher clarity (47–70% and >79%, respectively). In this study, the transmittance of the 1% untreated corn starch paste was 30%, slightly higher than reported values but still within the low-clarity category, possibly due to differences in starch purity or botanical source. Following AAT, GCWS samples prepared with NaOH contents above 4% all exhibited transmittance values exceeding 50% (Figure 3A), with some surpassing the clarity of potato starch. These findings demonstrate that AAT enables corn starch to acquire optical properties comparable to those of naturally high-clarity starches. Moreover, the transmittance of GCWS pastes remained relatively high after 7 days of storage. Statistical analysis indicated that NaOH content exerted the greatest influence on transmittance, with contribution rates of 50.8% for freshly prepared pastes and 52.7% after storage, underscoring the close relationship between ordered structure disruption and optical clarity. When NaOH content reached 4–6%, transmittance generally exceeded 70%, reflecting sufficient structural breakdown and reduced molecular entanglement. In contrast, GCWS prepared in 90% ethanol exhibited lower transmittance, consistent with restricted swelling and limited molecular expansion.

GCWS also displayed substantial viscosity at 35 °C (Figure 3B), confirming its cold-water solubility. However, viscosity did not increase simply with swelling or solubility but rather reflected a balance between viscosity enhancement due to swelling and viscosity reduction caused by granule breakdown. Viscosity was mainly affected by ethanol concentration, with contribution rates of 70.6% for viscosity at 35 °C and 57.1% for peak viscosity (PV). For instance, GCWS prepared in 50% and 70% ethanol at 50 °C exhibited high swelling factors, yet their PV values were lower than those prepared at 30 and 40 °C, likely due to excessive solubility and granule disintegration. GCWS prepared in 90% ethanol consistently showed extremely low viscosity, reflecting restricted swelling. This study investigated the influence of GCWS swelling in near-room-temperature water on solution viscosity, with results obtained under disruptive stirring conditions to approximate paste properties relevant to practical applications.

Taken together, comparisons across Figures 2 and 3 and the contribution rate analysis indicate that ethanol concentration plays a dominant role in regulating swelling and viscosity, whereas the NaOH content exerts a stronger influence on solubility and transmittance under high-temperature conditions. These findings highlight the multifaceted regulatory effects of AAT parameters on GCWS functional properties. Overall, AAT disrupts ordered structures while maintaining granule morphology, thereby producing GCWS with cold-water swelling, solubility, and transparency while retaining part of the pasting characteristics of native starch. Such properties not only overcome the limitations of conventional pregelatinized starch but also provide significant advantages in enhancing the convenience and sensory qualities of instant foods.

## Conclusion

This study confirms that alcoholic–alkali treatment (AAT) is a feasible and effective method for producing granular cold-water-soluble starch (GCWS) from corn starch. The results demonstrate that NaOH content is the key factor in structural disruption and functional transformation: levels below 2% are largely ineffective, whereas concentrations ≥5% consistently promote granule swelling and cold-water solubility. Ethanol concentration further modulates these effects: treatments in 50% and 70% ethanol enhance swelling, solubility, and transmittance, while 90% ethanol restricts molecular mobility and suppresses functional improvements. Temperature acts mainly as an intensifier, with structural changes most pronounced at 40–50 °C, though its influence is secondary compared with NaOH and ethanol.

The GCWS prepared in this study exhibited higher solubility at 25 °C than conventionally gelatinized starch. Its paste transmittance was more than 2.5-fold greater than that of untreated starch, and at 35 °C, the viscosity reached 73% of the peak viscosity of native starch, confirming its ability to retain paste consistency. These quantified outcomes establish a predictive framework: by adjusting NaOH, ethanol, and temperature, GCWS properties can be systematically designed to deliver targeted solubility, clarity, and viscosity. This framework not only provides a basis for food and industrial applications but also positions AAT as a scalable strategy for starch functionalization, capable of meeting diverse processing demands.

## Supplementary Material



## Notes

Supplementary Figures S1–S6: SEM and polarized-light micrograms of AAT starches treated with different treatment conditions (PDF)
