# Supplementary material for: Alcoholic–Alkali Modification of Corn Starch: A Framework for GCWS Design and Functional Tailoring
Source: ACS Omega. 2026 Aug 22;11(35):52703–13. doi: 10.1021/acsomega.6c03649 (PMC13563626; doi:10.1021/acsomega.6c03649)
Supplement: Supplementary file 1 [file ao6c03649_si_001.pdf]

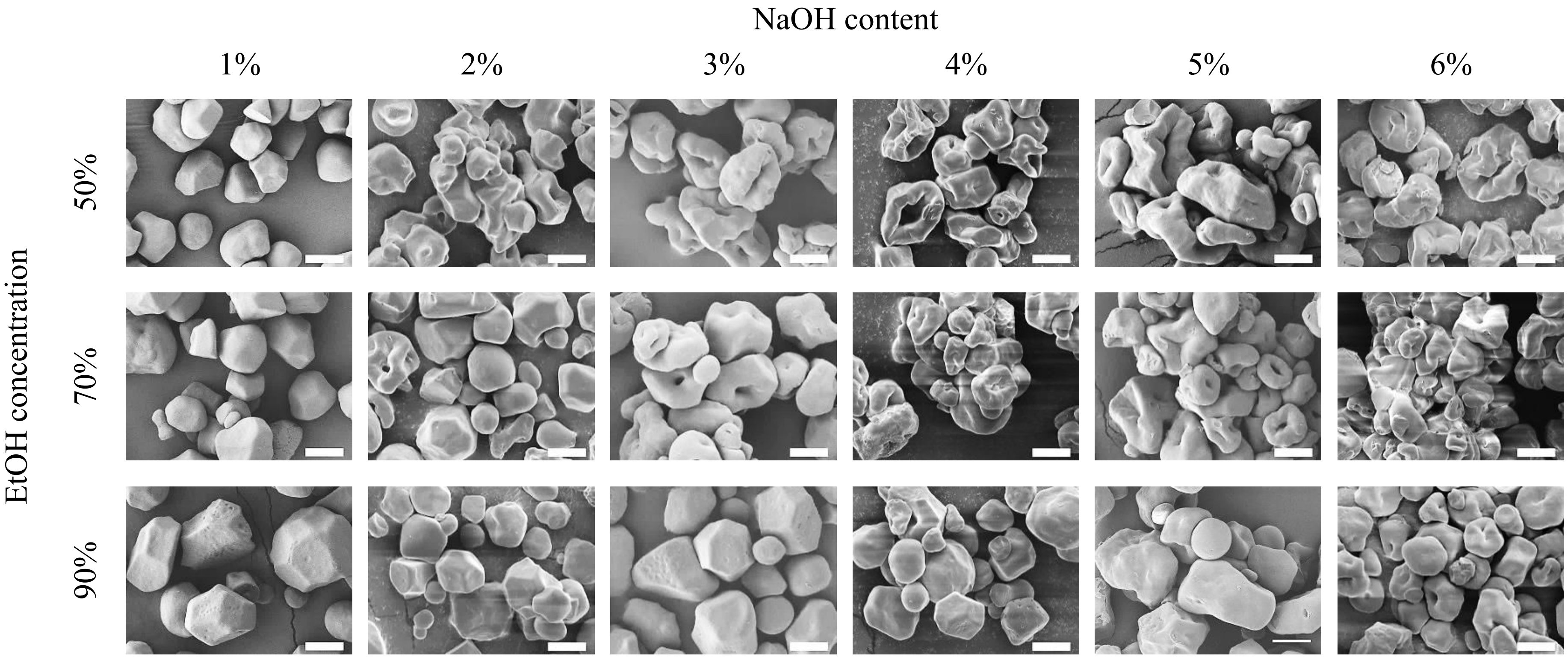

Supplementary Figure 1. Granule morphology observed by scanning electron micrography of corn starch after alcoholic-alkali treated at 30°C (bar = 10 μm).

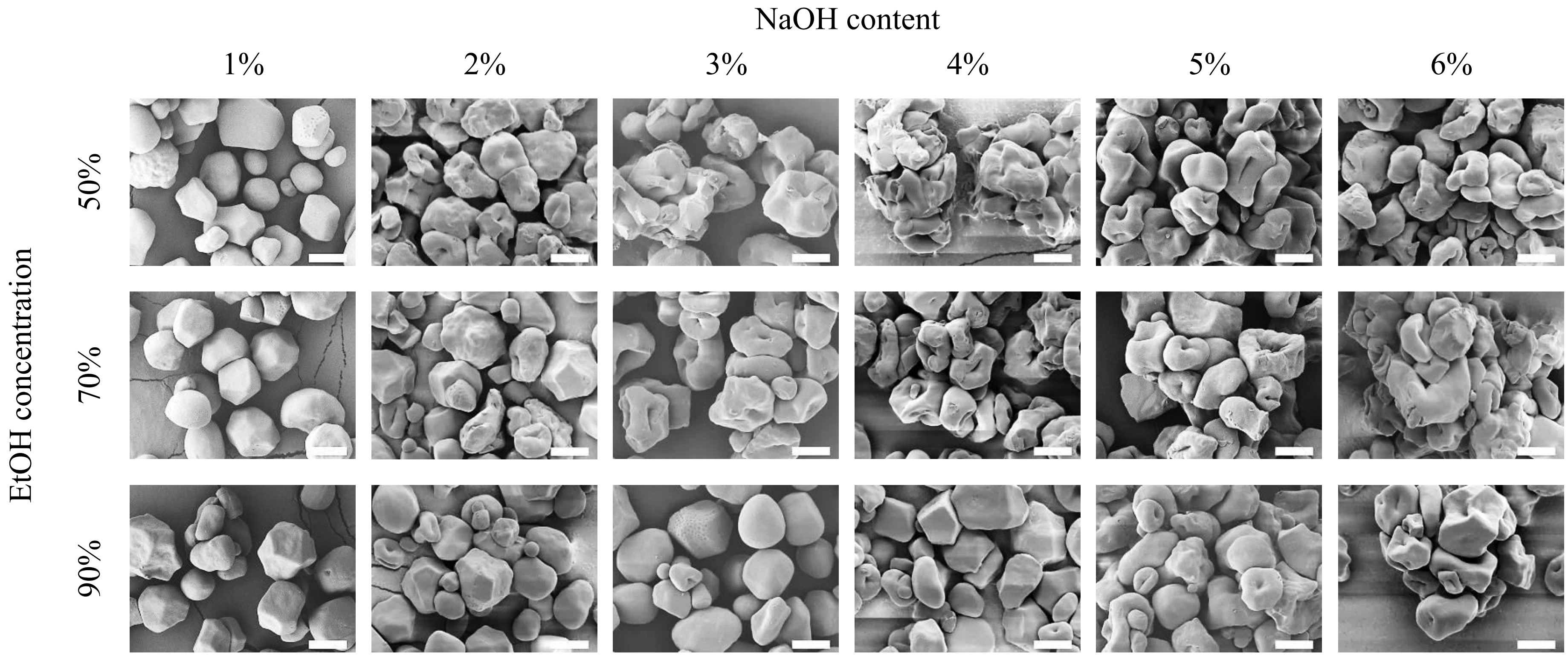

Supplementary Figure 2. Granule morphology observed by scanning electron micrography of corn starch after alcoholic-alkali treated at 40°C (bar = 10 μm).

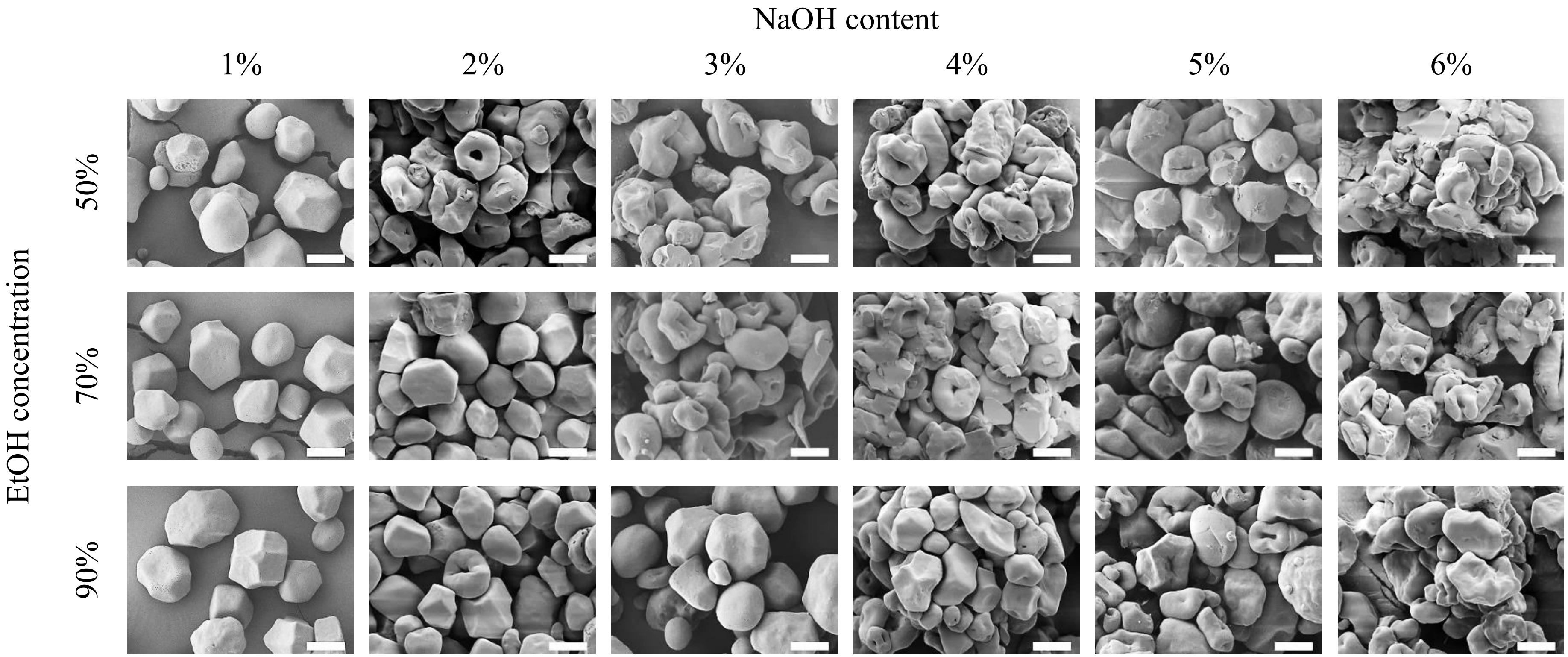

Supplementary Figure 3. Granule morphology observed by scanning electron micrography of corn starch after alcoholic-alkali treated at 50°C (bar = 10 μm).

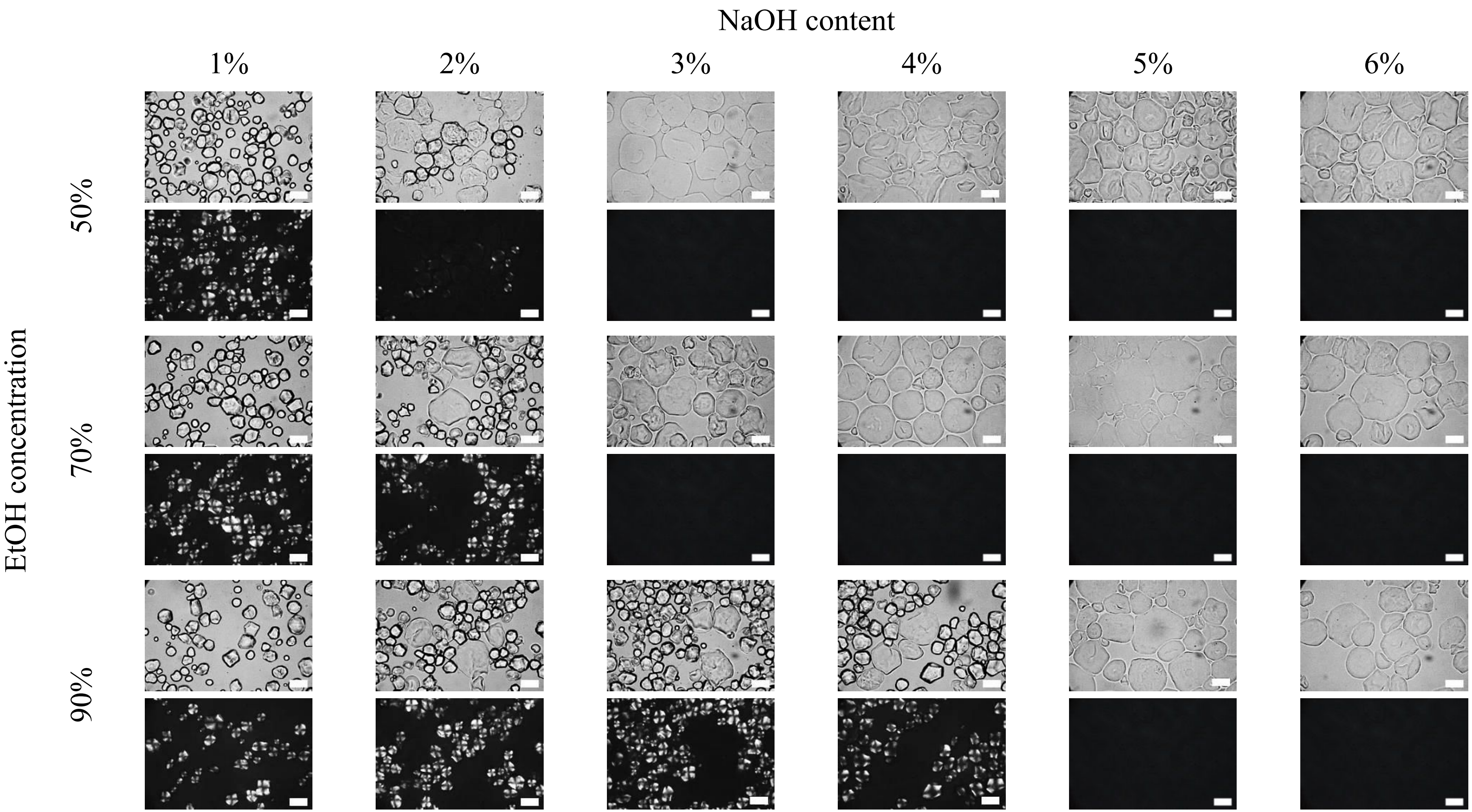

Supplementary Figure 4. Granule morphology and birefringence observed by light (LM) and polarized light (PLM) micrography of corn starch after alcoholic-alkali treated at 30°C (bar = 20 μm).

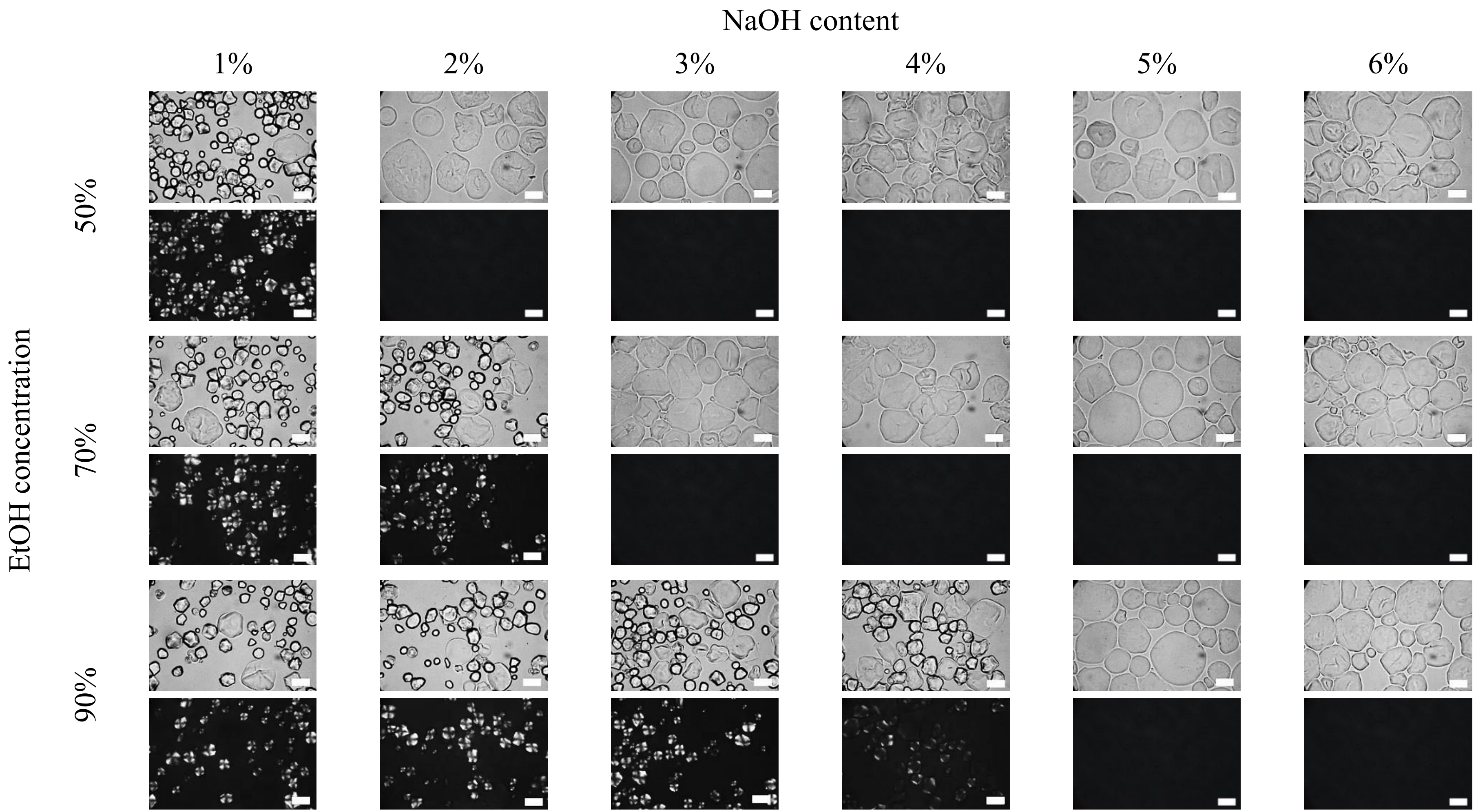

Supplementary Figure 5. Granule morphology and birefringence observed by light (LM) and polarized light (PLM) micrography of corn starch after alcoholic-alkali treated at 40°C (bar = 20 μm).

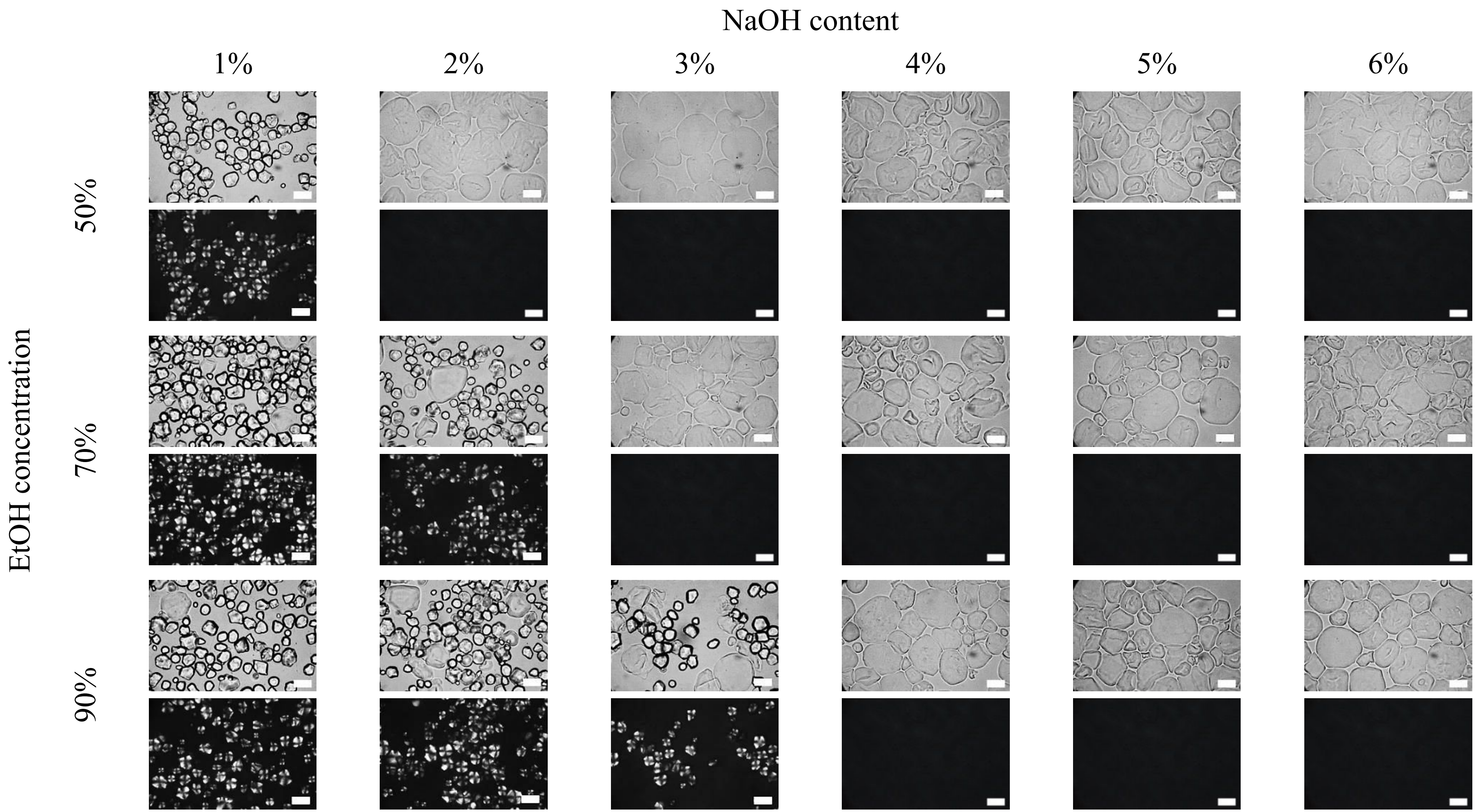

Supplementary Figure 6. Granule morphology and birefringence observed by light (LM) and polarized light (PLM) micrography of corn starch after alcoholic-alkali treated at 50°C (bar = 20 μm).
